# Digital twins in precision pharmacotherapy: emerging applications, challenges, and future directions

**DOI:** 10.3389/fdgth.2026.1881217

**Published:** 2026-08-18

**Authors:** Anan S. Jarab, Walid Al-Qerem, Hamza Jarab, Omar Jrab, Rahma Elsherif, Ahmad Z. Al Meslamani, Yazid Al Hamarneh, Salahdein Aburuz

**Affiliations:** 1 Department of Clinical Pharmacy, Faculty of Pharmacy, Jordan University of Science and Technology, Irbid, Jordan; 2 Department of Pharmacy, Faculty of Pharmacy, Al-Zaytoonah University of Jordan, Amman, Jordan; 3 Faculty of Medicine, Jordan University of Science and Technology, Irbid, Jordan; 4 Barts and the London School of Medicine and Dentistry, Queen Mary University of London, Zabbar, Malta; 5 College of Pharmacy, Al Ain University, Abu Dhabi, United Arab Emirates; 6 Department of Pharmacology, Faculty of Medicine and Dentistry, University of Alberta, Edmonton, AB, Canada; 7 Department of Pharmacology and Therapeutics, College of Medicine and Health Sciences, United Arab Emirates University, Al Ain, United Arab Emirates

**Keywords:** clinical pharmacy, digital twin, model-informed precision dosing, personalized medicine, pharmacodynamics, pharmacokinetics, precision pharmacotherapy

## Abstract

Digital twin technology, defined as dynamic digital models that represent individual patients, is emerging as a promising paradigm in precision pharmacotherapy. The integration of pharmacokinetic and pharmacodynamic (PK/PD) modeling, clinical data, genomic information, and real-time patient monitoring enables digital twins to shift drug therapy away from population-based averages toward individualized, adaptive decisions. This narrative review explores conceptual frameworks, emerging applications, methodological approaches, clinical value, limitations, and future directions of digital twins in pharmacotherapy, with particular emphasis on the role of clinical pharmacists. Unlike broader digital twin reviews that primarily emphasize technical architectures, disease-specific applications, or pharmaceutical research and development, this review focuses on the clinical-pharmacy translation layer: how digital twin outputs can be interpreted, validated, communicated, and converted into actionable medication decisions at the bedside and across ambulatory care settings. Key applications include precision dosing, polypharmacy management, antimicrobial stewardship, and the optimization of complex therapies, alongside important ethical, regulatory, and implementation challenges.

## Highlights

- Digital twins represent a dynamic, patient-specific modeling approach that enables individualized pharmacotherapy beyond population-based treatment strategies.
- By integrating pharmacokinetic/pharmacodynamic modeling with real-world clinical data, digital twins support adaptive drug dosing and real-time therapeutic optimization.
- Precision dosing of narrow-therapeutic-index drugs is one of the most immediate and clinically relevant applications of digital twin technology.
- Digital twins offer novel solutions for managing polypharmacy by simulating drug–drug interactions and supporting safer deprescribing decisions.
- Integration of patient-specific models into antimicrobial stewardship programs may reduce inappropriate antibiotic use while preserving clinical efficacy.
- Key barriers to implementation include data interoperability, model validation, explainability, and regulatory uncertainty.
- Ethical and equity considerations are essential to ensure digital twin models do not exacerbate existing disparities in medication outcomes.
- Future research should focus on prospective clinical trials, multi-omics integration, and population-level digital twin models to support public health decision-making.
- Clinical pharmacists are essential for turning digital twin predictions into practical medication decisions, including dose adjustment, monitoring plans, antimicrobial optimization, deprescribing, and toxicity prevention.
- The clinical value of digital twin-guided pharmacotherapy should be judged by the maturity of the evidence, from conceptual models and retrospective simulations to prospective studies showing meaningful patient outcomes.
- Existing model-informed precision dosing approaches, PBPK/QSP models, Bayesian dosing software, and pharmacometric platforms provide useful building blocks, but they should not be considered fully developed pharmacotherapy digital twins on their own.

## 1 Introduction

Pharmacotherapy is amongst the most widely used and significant strategies that have impacted modern healthcare practices (1). Medications have an essential role in the prevention, treatment, and cure of acute and chronic health problems. At the same time, the efficiency of drug-based therapy regimens varies significantly between individuals (2). While there have been significant advancements in the science of clinical pharmacology over the past several decades, the decision to adopt pharmacotherapy for treating a health condition is largely informed by population-based data obtained from randomized controlled trials and clinical practice guidelines (2). These methods are inadequate for accommodating variability in medication response based on physiologic, genetic, co-existing, concurrent, and environmental factors, to name a few (2).

Consequently, trial-and-error prescribing, dose adjustment after adverse drug reactions, and delayed attainment of therapeutic drug concentrations remain a significant problems (3, 4), which has been observed particularly for those agents with low therapeutic indices or complex pharmacokinetics, such as immunosuppressives, anticoagulants, antimicrobial agents, and many of those used in the oncology field (5). It has been noted that adverse drug events or ineffective use of medication is a major cause of problems for healthcare systems around the world and that this could be avoided (6).

In this review, precision pharmacotherapy refers to the individualized selection, dosing, monitoring, and adjustment of medication therapy according to patient-specific clinical, biochemical, genetic, and model-derived information (4, 7). This term is used to emphasize medication-use decisions in clinical practice, whereas precision pharmacology is broader and may also include pharmacological mechanisms, drug discovery, and preclinical or translational research. Established precision pharmacotherapy approaches include therapeutic drug monitoring, pharmacogenomic testing, and pharmacokinetic modeling, but these methods are often applied separately and may remain reactive rather than predictive (4).

Digital twin technology should be viewed as an extension of established patient-specific modeling approaches, including concentration-guided dosing and model-informed precision dosing, rather than as a completely separate paradigm (8). Its added value lies in linking these approaches with dynamic updating, longitudinal data integration, and prospective simulation of alternative treatment strategies (9, 10). Originally borrowed from engineering and systems science; a digital twin is a representation of a physical entity that is continuously updated using real-time data (11). In healthcare, this concept refers to a dynamic computational representation of an individual patient that is updated alongside the physical patient, reflecting changes in physiology, disease state, and therapeutic response (11–13). This enables clinicians to simulate potential interventions, predict treatment outcomes, and compare alternative strategies before applying them in clinical practice (11–13).

In the pharmacotherapy domain, a digital twin integrates pharmacokinetic/pharmacodynamic (PK/PD) models with longitudinal clinical and laboratory data, as well as multi-omics and sensor data, to make dynamical predictions about drug response and toxicities at the individual patient level (3, 9). The digital twins are continuously updated with new data and can be used for proactive, rather than reactive, medication management (3, 13).

As computational model technology, artificial intelligence, and health data infrastructure have matured, the feasibility of large-scale application of patient-specific simulation technology has increased (9, 14, 15). In addition, the changing role of clinical pharmacy practice has provided a new population of potential stakeholders in the development, validation, and implementation of digital twin technology, particularly as it pertains to pharmacotherapy-related decision-making (3, 11). Previous reviews have already discussed medical digital twins, virtual patients, and digital pharmacological twins in relation to personalized treatment simulation, in silico clinical trials, drug development, safety prediction, drug-drug interaction assessment, special populations, and clinical decision support (9–12, 16). However, less attention has been given to how these approaches can be translated into practical medication-related decisions within clinical pharmacy workflows. Therefore, this review focuses on the clinical-pharmacy role in interpreting digital twin outputs, identifying realistic pharmacist-facing use cases, and addressing the validation, governance, explainability, and accountability requirements needed before these tools can be safely integrated into routine pharmacotherapy.

This narrative review will address the rising role of digital twins in the era of precision pharmacotherapy, with a focus on clinical pharmacy practice. Its goal is to describe existing principles, investigate prospective applications, and identify certain problems and future prospects for research that could help move digital twin technology from experimental models to pharmaceutical practice.

## 2 Literature search strategy

This narrative review was based on a structured search of PubMed and Google Scholar for material published between January 2015 and May 2026. The timeline was chosen to highlight current advances in digital twin technology and its prospective applications in precision pharmacotherapy.

Search terms included combinations of the following keywords: “digital twin,” “digital pharmacological twin,” “precision pharmacotherapy,” “model-informed precision dosing,” “clinical pharmacy,” “pharmacokinetics,” “pharmacodynamics,” “physiologically based pharmacokinetic modeling,” “quantitative systems pharmacology,” “artificial intelligence in healthcare,” and “personalized medicine.” Boolean operators (AND/OR) were used to refine search strategies where appropriate.

Additional searches were conducted using related terms that reflect adjacent concepts and clinical implementation issues. These terms included “virtual patient,” “virtual population,” “in silico clinical trial,” “digital pharmacological twin,” “Bayesian dosing software,” “MIPD software,” “PBPK software,” “QSP model,” “drug-drug interaction prediction,” “pharmacogenomics,” “special populations,” “transplant pharmacotherapy,” “oncology precision dosing,” “antimicrobial stewardship,” “deprescribing,” and “clinical decision support.” Greater emphasis was placed on publications that linked computational modeling to medication selection, dose individualization, therapeutic drug monitoring, drug-drug interaction assessment, toxicity prediction, or pharmacist- and clinician-facing decision support.

Priority was given to peer-reviewed original research articles, methodological papers, systematic and scoping reviews, and position statements that addressed digital twin frameworks, pharmacometrics modeling, precision dosing applications, implementation challenges, and regulatory considerations in healthcare were prioritized.

Articles with no evident relation to medicine or healthcare and instead focused on engineering or industrial digital twin applications were excluded. Abstracts from conferences that did not provide full-text access were not prioritized unless they made significant conceptual advances.

Formal systematic review techniques, including a PRISMA flow diagram, quantitative synthesis, or predetermined study quality assessment, were not applied because this article was designed as a narrative review. Instead, the literature was synthesized thematically to identify major conceptual, methodological, clinical, and implementation issues relevant to digital twin-enabled pharmacotherapy. The included literature was organized into the following themes: conceptual digital twin frameworks, digital pharmacological twin models, model-informed precision dosing, clinical dosing software, drug- and disease-specific use cases, implementation barriers, regulatory considerations, and evidence maturity. This approach helped distinguish established pharmacometrics practice from emerging digital twin concepts and avoided overstating the current level of clinical implementation.

### 2.1 Positioning the review within the existing literature

Digital twins and related patient-specific modeling approaches have already been discussed in several areas of healthcare and pharmacology. Broad reviews have described the use of digital twins in diagnosis, treatment planning, chronic disease monitoring, care coordination, hospital operations, in silico clinical trials, and drug discovery (12, 16). Other recent work has also helped define the structure of a medical digital twin, including the patient, data connection, patient-in-silico model, clinical interface, and synchronization between the real patient and the virtual model (10). In pharmacology, the concept of digital pharmacological twins has been proposed to integrate PK/PD modeling, mechanistic modeling, artificial intelligence, multi-omics, clinical information, and environmental data to support precision medicine (9).

Therefore, this review does not suggest that digital twins have been absent from pharmacotherapy research. Rather, its focus is more specific: how digital twin outputs can be translated into medication-related decisions in clinical pharmacy practice. This includes how pharmacists can verify model inputs, judge whether predictions are clinically actionable, interpret uncertainty, document recommendations, and apply digital twin information in therapeutic drug monitoring, antimicrobial stewardship, oncology pharmacy, transplant pharmacotherapy, pharmacogenomics, polypharmacy review, and deprescribing. By focusing on the medication-use process, this review aims to clarify the role of clinical pharmacists as interpreters, validators, and implementers of digital twin-guided pharmacotherapy.

## 3 Conceptual framework of digital twins in pharmacotherapy

Digital twins in pharmacotherapy are “computational models that are uniquely patient-specific, dynamic, continuously evolving representations of individual patients” (9). Thus, unlike other computational or static model-based approaches, digital twins maintain a dynamic relationship with the individual, giving and taking input, where clinical data from real individuals are used to update the model, while the model, in turn, provides predictions that can aid in decision-making in pharmacotherapy (9).

At the core of a pharmacotherapy-focused digital twin lies a mechanistic or semi-mechanistic modelling framework, most commonly pharmacokinetic and pharmacodynamic (PK/PD) models (3, 9). PK/PD modelling describes drug absorption, distribution, metabolism, and excretion, as well as drug exposure and effect (17). In more advanced systems, physiologically based pharmacokinetic modeling or quantitative systems pharmacology modeling is employed to consider organism-level physiological phenomena and biological processes (3, 9). In addition, patient-specific parameters including age, body composition, renal and liver function, inflammation, and disease severity are included in the model to individualize the simulation (3, 9).

Clinical data streams play an important role in ensuring the model remains relevant and accurate. Longitudinal laboratory values, therapeutic drug monitoring, vital signs, and clinical end points enable the model to be recalibrated throughout the progression of the patient's disease (9, 10). To improve predictions, more data sources are being investigated, including pharmacogenomic profiles, wearable sensor data, and patient-reported outcomes (9, 10, 18). The integration of these various data types allows the digital twin to reflect both short-term physiological fluctuations and long-term health status changes.

Because digital twin predictions are derived from mechanistic and computational models, they are frequently expressed with associated uncertainty measures, allowing clinicians to interpret simulated outcomes probabilistically rather than as deterministic outputs (19). A defining advantage of digital twins in pharmacotherapy is their capacity for prospective simulation. Clinicians and pharmacists can explore with “what-if” scenarios, such as adjusting a dose, changing dosing intervals, adding or discontinuing a medication, or switching therapies. The digital twin can predict how these changes will affect drug exposure, efficacy, and toxicity before they are administered to the patient (9). This predictive capability enables proactive decision-making while reducing reliance on trial-and-error methods.

There are major differences between digital twins and conventionally employed rule-based alerting and static dosing tools. These discrepancies are mostly focused on how digital twins may learn from fresh data, which affects their ability to change their predictions over time and match the patient's changing conditions to the recommendations (9, 20). For instance, during complex pharmacotherapy management, which involves critical illness, chronic disease evolution, and polypharmacotherapy, the patient's variable physiological status requires an application tool that can update its recommendations intelligently (9).

**Figure 1:**
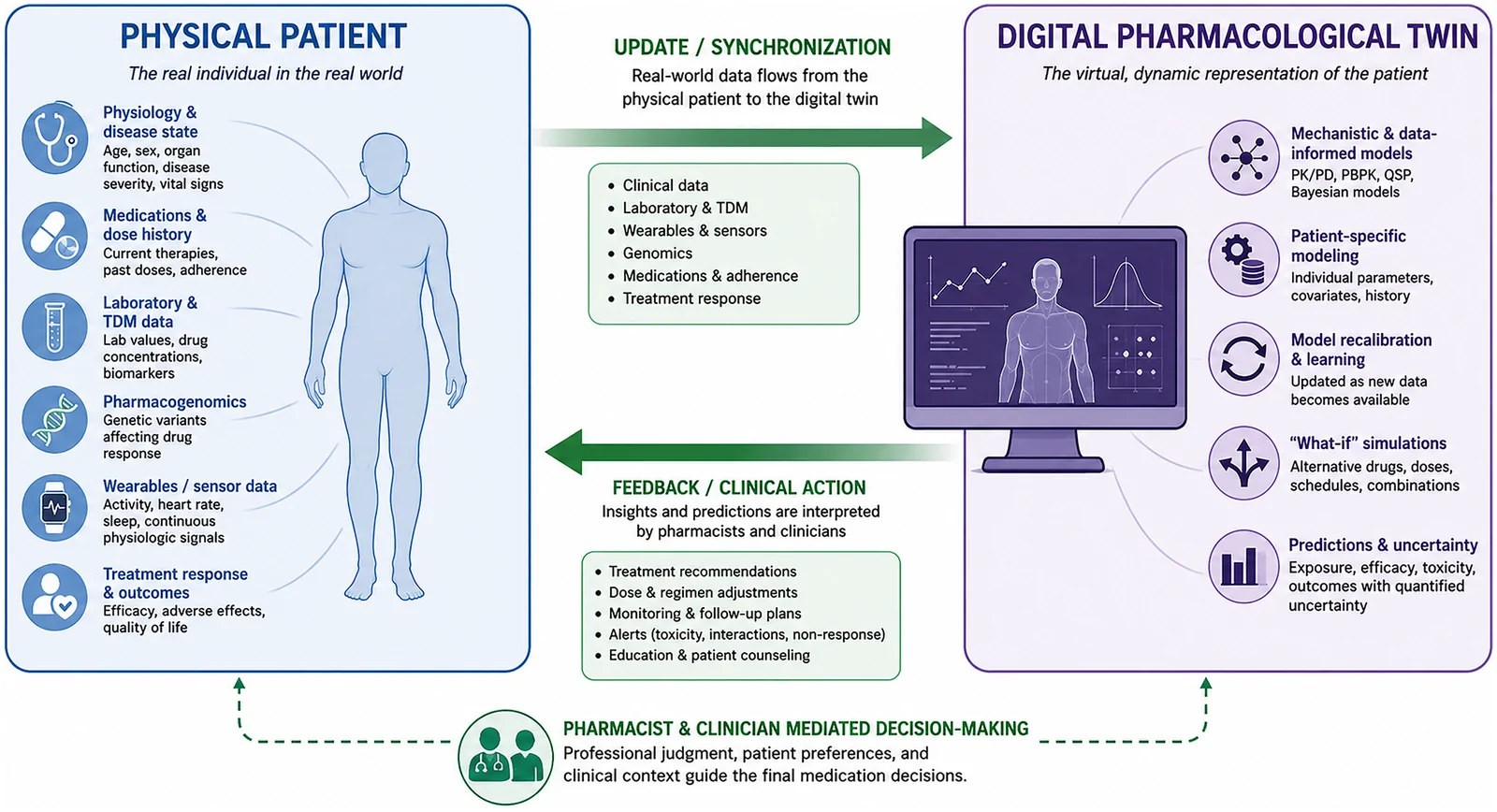
.Digital twin–enabled precision pharmacotherapy linking patient data, virtual modeling, and pharmacist-clinician decision-making.

Taken together, these traits establish digital twins as a concept rather than a simple field. They use traditional pharmacometric methodologies in conjunction with data science developments to provide tailored representations of medication response (9). In that respect, digital twins provide a theoretical foundation for moving precision pharmacotherapy beyond reactive dose modification and toward predictive, patient-centered medication management. A conceptual overview of the structure, data inputs, and clinical decision pathways of digital twin-enabled precision pharmacotherapy is presented in Figure 1.

## 4 Digital twins vs. Model-Informed precision dosing (MIPD)

Model-informed precision dosing (MIPD) is a quantitative pharmacological method that customizes medication regimens using mathematical PK/PD models. MIPD commonly uses Bayesian forecasting applied to population-based PK models and may incorporate patient-specific covariates, biomarkers, and therapeutic drug monitoring data when available (21). Although digital twins and MIPD share fundamental ideas, significant conceptual differences must be emphasized. MIPD's primary goal is to optimize medication dose using pharmacokinetic models, generally Bayesian, informed by TDM and specified patient variables (5, 21). Its focus is often drug-specific and episodic, with model changes taking place at predetermined clinical time points.

Concentration-guided dosing is closely related to MIPD and provides an important historical and conceptual foundation for individualized dose prediction. It uses measured drug concentrations, pharmacokinetic principles, and patient-specific information to interpret exposure and guide individualized dosing decisions (8). In this review, digital twins are therefore not presented as replacements for concentration-guided dosing or MIPD, but as broader systems that may incorporate these approaches while adding repeated synchronization with longitudinal patient data, simulation of alternative therapy scenarios, explicit uncertainty communication, and integration of medication response with disease trajectory, interacting drugs, organ function, and clinical outcomes (8–10).

Digital twins may integrate multimodal data streams, including wearable-sensor, pharmacogenomic, laboratory, electronic health-record, and real-world outcome data, depending on their intended clinical use (10, 11). Digital twins attempt to replicate system-level treatment responses, multi-drug interactions, disease progression, and potential “what-if” situations rather than just concentrating on exposure measurements for a single medication (10, 11). Repeated or continuous updating, bidirectional information exchange, uncertainty quantification, and representation of changing physiological and pharmacological states throughout time are some of its distinguishing features (11).

Nonetheless, the distinction between advanced MIPD platforms and early digital twin systems may be blurred. MIPD can be considered one of the closest clinically available foundations for pharmacotherapy-focused digital twins, rather than a separate approach (5, 21, 22). In current practice, MIPD platforms already use population pharmacokinetic models, Bayesian forecasting, patient covariates, therapeutic drug concentrations, and exposure targets to support individualized dosing (21–23). However, a system should not be considered a pharmacotherapy digital twin simply because it estimates an individualized dose. A digital twin requires a broader and more dynamic representation of the patient, repeated updating with new clinical data, clear communication of uncertainty, the ability to compare alternative treatment scenarios, and integration of medication response with disease progression, organ function, drug-drug interactions, pharmacogenomic information, and clinical outcomes. Therefore, the most realistic near-term pathway is not to replace MIPD, but to build on validated MIPD workflows and extend them into dynamic, multimodal, pharmacist-interpretable digital twin systems. Key conceptual differences between model-informed precision dosing and digital twins are summarized in Table 1.

**Table 1:** Typical distinctions and areas of overlap between model-informed precision dosing and pharmacotherapy digital twins.

Feature	Model-informed precision dosing	Pharmacotherapy digital twin
Primary objective	Individualize a dosing regimen to achieve a predefined exposure, efficacy, or toxicity target.	**Maintain a patient- or subsystem-specific representation** to predict treatment response, **compare interventions**, and support clinical decisions.
Scope	Usually focused on a particular drug, regimen, or exposure–response relationship, although multiple covariates, biomarkers, and interactions may be included.	**Potentially broader and multiscale**, linking medication response with physiology, disease progression, interacting treatments, and clinical outcomes. It may represent **an organ, disease, biological system, or broader patient state** rather than the entire patient.
Data sources	Population PK/PD or other pharmacometric models, patient covariates, dosing history, drug concentrations, biomarkers, pharmacogenomic information, and clinical data where relevant. TDM is common but not mandatory in every application.	**Longitudinal, intended-use-specific data** that may include EHR information, laboratory results, PK/PD data, imaging**, omics, environmental information, wearable measurements**, and clinical outcomes. Not every twin must include every data type.
Uncertainty	Pharmacometric models characterize population and residual variability; Bayesian approaches may estimate patient-specific posterior or predictive uncertainty, although this is not always fully propagated or communicated in clinical software.	**Verification, validation, and uncertainty quantification** should address uncertainty across **integrated data sources, model components, calibration, updating, and predictions**, although current digital-twin implementations do not always achieve this fully.
Model updates	Re-estimated when new concentrations, covariates, biomarkers, or clinical observations become available; updates may be occasional, repeated, or near real time.	**Dynamically synchronized with the physical patient over time.** Updating may be periodic or continuous depending on the intended use and availability of clinically meaningful data.
Simulation style	Compares alternative doses, intervals, infusion strategies, or monitoring plans to achieve medication-specific targets.	May compare **broader treatment alternatives, combinations, downstream physiological effects, and possible disease or health trajectories**.
Feedback loop	An iterative, usually clinician-mediated dosing-monitoring-adjustment loop focused on a medication or regimen.	**A broader bidirectional loop** in which patient data update the virtual representation and model predictions inform clinician-mediated interventions and subsequent monitoring. Automatic control is not required.

Bold text indicates the category headings used to organize the comparison.

## 5 Clinical pharmacy use cases in precision pharmacotherapy

Digital twins are most useful in clinical pharmacy when they are linked to clear medication decisions rather than described only as a broad technological concept. The most realistic early applications are likely to be high-risk therapies where drug exposure can be measured or estimated, dose adjustment is clinically meaningful, and pharmacists already have an established role in monitoring and decision-making (7, 22).

Because fully integrated pharmacotherapy digital twins are not yet routine clinical tools, these examples are framed as near-term clinical use cases and current modeling foundations rather than mature digital twin deployments.

### 5.1 Narrow-therapeutic-index drugs and therapeutic drug monitoring

Narrow-therapeutic-index drugs are among the most practical early targets for digital twin-guided pharmacotherapy because small changes in exposure can lead to toxicity or treatment failure (7, 22). Examples include vancomycin, aminoglycosides, tacrolimus, busulfan, voriconazole, phenytoin, digoxin, and selected anticancer drugs, many of which are already managed through therapeutic drug monitoring and pharmacist-led dose adjustment (22, 24).

Vancomycin illustrates how current practice could evolve toward a digital twin workflow. Current guidelines recommend AUC-guided monitoring, preferably using Bayesian estimation, rather than relying only on trough concentrations (24). A pharmacist-facing digital twin could extend this approach by combining dose history, sampling time, renal function, infection severity, MIC assumptions, and exposure to nephrotoxic drugs to support individualized dosing and monitoring decisions (9, 24). However, the pharmacist would still need to confirm the accuracy of the clinical data, assess whether the model assumptions fit the patient, and decide whether the recommendation is safe and clinically reasonable (19).

Similar principles apply to other high-risk therapies such as tacrolimus and busulfan, where exposure variability, toxicity risk, and the need for careful monitoring already make pharmacist involvement essential (25, 26). These examples show that digital twins are most likely to develop first by building on established therapeutic drug monitoring and MIPD workflows, rather than replacing them (19, 22).

### 5.2 Polypharmacy, drug–drug interactions, and deprescribing

Digital twins may also support polypharmacy management because medication-related harm often results from the combined effects of multiple drugs, comorbidities, organ dysfunction, and patient-specific risk factors (9, 20). A pharmacotherapy digital twin could combine the medication list, dosing schedule, renal and hepatic function, pharmacogenomic phenotype, laboratory trends, frailty, bleeding risk, QTc interval, anticholinergic burden, adherence, and patient goals of care (9, 20).

In this setting, the pharmacist's role would be to translate predicted risk into a practical medication plan. For example, if a model predicts increased anticoagulant exposure after the addition of a strong P-glycoprotein or CYP3A4 inhibitor, the pharmacist must decide whether dose adjustment, temporary interruption, therapeutic substitution, laboratory monitoring, or patient counseling is the safest response (27). PBPK models are already used to predict or explain drug-drug interactions, but continuously updated patient-specific interaction twins are not yet routine clinical tools (9, 27). Therefore, this use case should be presented as promising but still requiring prospective validation using outcomes such as bleeding, falls, arrhythmia, hospitalization, and preventable adverse drug events (16, 19).

### 5.3 Antimicrobial stewardship and infection pharmacotherapy

Antimicrobial stewardship is another strong use case because antibiotic decisions must account for both patient pharmacokinetics and pathogen pharmacodynamics (28). A stewardship-oriented digital twin could integrate renal function, body size, critical illness physiology, infection source, pathogen susceptibility, MIC values, drug concentrations, local resistance patterns, and toxicity risk (28).

For clinical pharmacists, this approach could support dose selection, infusion strategy, repeat drug-level timing, de-escalation, and toxicity monitoring (24, 28). It may be particularly useful for vancomycin, aminoglycosides, and beta-lactams in patients with acute kidney injury, renal recovery, augmented renal clearance, or critical illness (24, 28). However, most antimicrobial digital twin applications remain closer to simulation, feasibility testing, or early translational work than routine clinical implementation (16, 19).

### 5.4 Oncology pharmacy and exposure-guided treatment

Oncology pharmacy is a priority area because many anticancer therapies have narrow therapeutic windows, high interpatient variability, pharmacogenomic determinants, and serious consequences of both toxicity and treatment failure. Digital twins could support exposure-guided treatment by combining organ function, pharmacogenomics, prior therapy, drug concentrations when available, toxicity trends, tumor response, and patient-reported outcomes (9, 29).

Model-informed dose optimization in oncology has already been prospectively evaluated for selected drugs, but it is not yet widely integrated into routine care (29). A recent review identified 16 oncology drugs with prospective MIPD validation or implementation studies (29). For 5-fluorouracil, IATDMCT recommendations strongly support therapeutic drug monitoring in colorectal and head-and-neck cancer because body-surface-area dosing does not adequately reduce exposure variability (30). These examples suggest that oncology has important building blocks for digital twin–guided pharmacotherapy, although full oncology digital twins would still need stronger integration of tumor dynamics, toxicity prediction, adherence, and patient-centered outcomes (29).

### 5.5 Special populations

Digital twins may be especially useful in populations that are underrepresented in clinical trials, including neonates, children, pregnant patients, older adults with frailty, patients with obesity, patients with renal or hepatic impairment, and critically ill patients receiving organ support (7, 22). In these groups, standard dosing is often extrapolated from populations with different physiology, which increases uncertainty around exposure and response (7, 22).

For pharmacists, the key question is whether the patient is represented by the model being used. A recommendation developed in stable adults should not be applied uncritically to a neonate, a pregnant patient, a patient receiving extracorporeal membrane oxygenation, or a patient with rapidly changing renal function (7, 22). Digital twins for special populations should therefore make uncertainty, assumptions, missing data, and extrapolation clear to the clinician (19). This use case is clinically important, but it should be presented cautiously because representativeness, model credibility, and prospective outcome validation remain major barriers (16, 19).

Together, these applications illustrate how digital twins may move precision pharmacotherapy from reactive dose adjustment toward proactive, simulation-informed medication management (9, 19).

## 6 Methodological challenges

Despite the promise of digital twins in pharmacotherapy, certain methodological challenges need to be addressed. First, the issue of data quality and integration is a major barrier for the development of high-fidelity digital twins, which demands the integration of data from different sources such as electronic health records, laboratory tests, genomics, wearables, and others (31, 32). Missing data, inconsistent measurement units, and heterogeneity in data formats can reduce model accuracy and limit generalizability (32).

Second, model creation and validation provide considerable problems. Mechanistic PK/PD, PBPK, and QSP models must be calibrated and validated using real-world data, such as therapeutic target achievement and adverse events (31, 32). The intricacy of these models may diminish transparency and interpretability, thus limiting therapeutic use (32). Approaches such as sensitivity analysis, uncertainty quantification, and iterative feedback loops are required to assure reliability and reproducibility (32). However, standardized validation frameworks and performance benchmarks for digital twins in pharmacotherapy remain poorly defined, complicating cross-study comparison and regulatory evaluation.

Third, ethical, privacy, and equity concerns are prevalent. Digital twins rely on sensitive patient information, raising issues of consent, data security, and potential misuse (20). Models trained on non-representative datasets may perpetuate health inequities or provide misleading predictions. Thus, strict ethical frameworks and governance standards are required for responsible deployment.

Finally, computational and technological hurdles arise. Processing multimodal data, conducting complicated simulations in near real time, and incorporating predictions into clinical processes need a strong infrastructure, scalable software platforms, and interoperability standards (20). Existing tools already support parts of this process, including PBPK/QSP platforms such as PK-Sim/MoBi, pharmacometric modeling environments such as NONMEM and Monolix, and Bayesian dosing platforms such as InsightRx, DoseMeRx, MWPharm, and TDMx (22, 23). However, these tools should be viewed as building blocks rather than complete pharmacotherapy digital twins, because clinical use still depends on validation, EHR integration, usability, workflow fit, and evidence of patient benefit (5).

## 7 Role of the clinical pharmacist

Clinical pharmacists are the link between model prediction and safe medication use, because a digital twin output should not be treated as a medication order by itself (20). Before a recommendation is used, pharmacists verify the indication, medication history, dose administration record, timing of drug levels, organ function, key laboratory values, pharmacogenomic results, interacting medicines, adherence concerns, and whether the patient fits the model's intended population (22). During interpretation, they assess whether the recommendation is clinically plausible, whether the uncertainty is acceptable, and whether the suggested action fits treatment goals, guidelines, formulary restrictions, and patient-specific risks (20).

After implementation, pharmacists monitor drug exposure, treatment response, toxicity, adherence, and patient-centered outcomes, and they document the model inputs, assumptions, recommendation, monitoring plan, prescriber communication, and observed response (19, 33). These follow-up data are important for identifying unsafe extrapolation, workflow problems, bias, or model failure over time (16, 19). In this way, digital twin implementation extends pharmacists' existing roles in therapeutic drug monitoring, antimicrobial stewardship, pharmacogenomics, medication therapy management, and medication safety rather than creating a separate parallel workflow (22, 33).

Clinical pharmacists are essential for the successful development, implementation, and application of digital twins in pharmacotherapy. Their understanding of pharmacokinetics, pharmacodynamics, medication safety, and therapeutic drug monitoring allows them to validate model assumptions, evaluate predictions, and turn outputs into tailored prescription plans Silva and Vale (20),. Furthermore, clinical pharmacists are well-positioned to provide governance and supervision for digital twin-guided pharmacotherapy, ensuring that model outputs are used appropriately and within established clinical and safety standards.

Pharmacists can also inform model refinement by providing clinical feedback, with real-world outcomes that can update and refine predictive accuracy. By integrating computer predictions with clinical judgment, pharmacists leverage digital twin insights to prevent drug-drug interactions, optimize dose, and minimize adverse effects in polypharmacy and complex medical conditions. They also educate multidisciplinary teams and patients about the use of predictive models, ensuring the safe and effective implementation of digital twin-guided medication (12, 33). Evidence from UAE community pharmacy practice suggests that pharmacists are increasingly aware of advanced technological tools, including robotic systems, but remain concerned about workflow integration, implementation barriers, and the need for adequate training and awareness programs (34). The safe integration and long-term sustainability of digital twin technologies in clinical practice are supported by their participation in protocol development, outcome monitoring, and ongoing feedback.

By bridging computational outputs and clinical practice, clinical pharmacists help ensure that digital twins are not only theoretically accurate but also actionable in real-world patient care, enhancing precision, safety, and therapeutic outcomes.

## 8 Implementation and regulatory challenges

Adoption of digital twins in clinical practice requires integration into existing healthcare systems without disrupting the workflow, and electronic health record (EHR) interoperability is vital to ensure seamless data flow into and out of digital twin platforms. However, many healthcare systems lack standardized data formats or adequate interfaces (35). Moreover, computational predictions should be delivered in a clinician-friendly manner, which means they are transparent, interpretable, and provide practical recommendations.

Depending on their intended purpose and level of autonomy, digital twins may be classified as medical devices, software as a medical device (SaMD), or clinical decision-support aids by regulatory authorities (36). Regulatory authorities such as the FDA and EMA need validation of prediction performance, safety, and reliability (37). A tool that only displays predicted drug exposure for clinician review may require a different level of oversight than a system that recommends a specific dose, prioritizes one therapy over another, or updates its recommendations as new patient data become available (36). For adaptive models, developers should define the intended-use population, required data inputs, model-updating process, human-override options, monitoring procedures, and post-deployment surveillance plan (19). Post-market surveillance, periodic recalibration, and adherence to privacy standards such as HIPAA or GDPR are all essential to ensure ongoing patient safety (37).

Implementation also raises organizational and educational concerns. Clinicians and pharmacists must be trained to understand the assumptions, limitations, and appropriate application of digital twin outputs (16, 38). Similar concerns have been reported for large language models in UAE community pharmacies, where pharmacists identified human supervision, insufficient training, cybersecurity risks, technical failures, and limited pharmacy-specific AI tools as key barriers to implementation (39). Multidisciplinary collaboration among computer scientists, pharmacists, and physicians is essential to effectively integrate these tools into patient care while maintaining clinical control.

Finally, cost and resource constraints might impact scalability. Adoption in resource-constrained healthcare settings may be limited by the infrastructure, computational capacity, and staff skills required for developing, verifying, and maintaining patient-specific models (16).

## 9 Current status of digital twins in pharmacotherapy

At present, digital twin-guided pharmacotherapy should be considered an emerging translational approach rather than an established standard of care (16). The strongest clinical foundation comes from related patient-specific dosing practices, including concentration-guided dosing, therapeutic drug monitoring, and model-informed precision dosing, where measured concentrations and patient covariates are used to individualize drug exposure (8, 22). Examples such as AUC-guided vancomycin monitoring and established TDM/MIPD workflows for tacrolimus and busulfan show how model-based individualized dosing can be incorporated into clinical care, but these workflows should be viewed as foundations for pharmacotherapy digital twins rather than complete digital twin systems (24–26).

Most pharmacotherapy-related digital twin applications are still supported by early-stage evidence, including conceptual frameworks, simulation studies, virtual cohorts, pilot studies, and selected model-informed dosing workflows (10) External validation against observed drug concentrations, biomarkers, adverse events, or clinical outcomes is an important step, but it is not the same as proving clinical benefit (19). Similarly, surrogate pharmacometric endpoints, such as target concentration attainment, AUC-based exposure metrics, or modeled toxicity risk, are useful for evaluating dosing logic but should not be treated as direct evidence of improved patient outcomes (10). Therefore, future studies should move beyond prediction accuracy and evaluate whether digital twin-guided pharmacotherapy reduces adverse drug events, treatment failure, hospitalization, mortality, workflow burden, and cost while improving patient-centered outcomes. Prospective, adequately powered clinical trials are still needed before these tools can be recommended for broad routine use in pharmacotherapy (10, 16, 19).

Overall, the current evidence supports feasibility and clinical rationale, but not routine implementation of complete pharmacotherapy digital twins. Broader adoption will require prospective, adequately powered studies, standardized validation methods, interoperable data systems, transparent uncertainty reporting, and regulatory guidance.

## 10 Future directions

There are promising prospects for digital twin technology applied to pharmacotherapy and several important research and development priorities. Prospective clinical trials are needed to evaluate the impact of digital twin-guided therapy on patient outcomes such as time-to-therapeutic target, adverse events, and cost-effectiveness (16). The integration of multi-omics data, which includes genomes, proteomics, metabolomics, and microbiome data, has the potential to improve personalized predictions and help precision medicine projects (9). Simultaneously, the development of standardized data models, frameworks of interoperability, and validation approaches will be important for the scalable replication of the deployment of digital twins (16).

Advancements in machine learning and artificial intelligence will improve the digital twin's power to learn from emerging data streams, forecast clinical deterioration, and recommend improved treatments in near real time (15, 40). Population-level simulations can also help with public health decision-making by predicting illness development, therapeutic response, and resource allocation across patient groups (40).

Ethical issues, equity, and governance will be important to address. To avoid worsening health inequities, it is critical that digital twins be built using representative data, data privacy, and accessibility to various groups in the future (40).

Future studies should focus on medication-use outcomes that are meaningful to both clinical pharmacists and patients. These outcomes include time to therapeutic target, preventable adverse drug events, treatment failure, nephrotoxicity, bleeding, transplant rejection, antimicrobial de-escalation, successful deprescribing, adherence, patient understanding, workflow burden, alert fatigue, and cost-effectiveness. Clinical pharmacists should be involved in study design from the beginning to help define clinically useful outputs, acceptable levels of uncertainty, documentation requirements, and escalation pathways when model predictions differ from clinical judgment. Pragmatic studies embedded in antimicrobial stewardship programs, transplant clinics, oncology services, and therapeutic drug monitoring services may provide the most practical evidence for real-world implementation (10, 16).

Finally, closer collaboration between industry, regulatory agencies, healthcare providers, and clinical pharmacists will be essential to translate digital twin technology from experimental platforms into scalable, reliable, and safe clinical tools that improve pharmacotherapy outcomes (40).

## 11 Review limitations

This review may be subject to publication bias, as studies with positive findings are more likely to be published. Inclusion of access to full-text or open-access articles was also limited, which might have narrowed the completeness of the review. Variability in the health-care system, medication, and digital support between areas may also have an impact on the data overall generalizability. Despite these limitations, the review provides a complete, evidence-based assessment of the current state, applications, and limitations of digital twin technology in precision pharmacotherapy, highlighting areas for future research, clinical, and policy applications.

## 12 Conclusion

Digital twin technology represents an emerging translational framework for precision pharmacotherapy rather than a proven replacement for established clinical judgment, therapeutic drug monitoring, or pharmacist-led medication by combining mechanistic PK/PD models with real-world clinical, laboratory, and multi-omics data, digital twins enable clinicians and pharmacists to simulate therapeutic interventions, optimize dose, predict adverse events, and manage complex polypharmacy. Despite methodological, technical, ethical, and regulatory barriers, digital twins have enormous potential for improving patient outcomes, safety, and cost-effectiveness. Clinical pharmacists may play an essential role in model validation, prediction interpretation, and intervention implementation using digital twins. Future research should prioritize prospective clinical trials, the incorporation of multi-omics and sensor data, machine learning-based modifications, and the creation of fair, open governance frameworks to ensure safe and widespread deployment of digital twin technologies in healthcare. As the field advances, the clinical value of pharmacotherapy digital twins will depend less on technological sophistication alone and more on prospective validation, transparent uncertainty communication, equitable data representation, workflow integration, and the ability of pharmacists and other clinicians to translate predictions into safe, patient-centered medication decisions.
